# Natural history of prostate cancer on active surveillance: stratification by MRI using the PRECISE recommendations in a UK cohort

**DOI:** 10.1007/s00330-020-07256-z

**Published:** 2020-09-30

**Authors:** Francesco Giganti, Armando Stabile, Vasilis Stavrinides, Elizabeth Osinibi, Adam Retter, Clément Orczyk, Valeria Panebianco, Bruce J. Trock, Alex Freeman, Aiman Haider, Shonit Punwani, Clare Allen, Alex Kirkham, Mark Emberton, Caroline M. Moore

**Affiliations:** 1 Department of Radiology, University College London Hospital NHS Foundation Trust, London, UK; 2 Division of Surgery & Interventional Science, University College London, 3rd Floor, Charles Bell House, 43-45 Foley St., London, W1W 7TS UK; 3 Department of Urology and Division of Experimental Oncology, Vita-Salute San Raffaele University, Milan, Italy; 4 Department of Urology, University College London Hospital NHS Foundation Trust, London, UK; 5 Department of Radiological Sciences, Oncology and Pathology, Sapienza University of Rome, Rome, Italy; 6 The James Buchanan Brady Urological Institute and Department of Urology, Johns Hopkins University School of Medicine, Baltimore, MD USA; 7 Department of Pathology, University College London Hospital NHS Foundation Trust, London, UK; 8 Centre for Medical Imaging, University College London, London, UK

**Keywords:** Urogenital neoplasms, Prostatic neoplasms, Magnetic resonance imaging, Biopsy

## Abstract

**Objectives:**

The PRECISE recommendations for magnetic resonance imaging (MRI) in patients on active surveillance (AS) for prostate cancer (PCa) include repeated measurement of each lesion, and attribution of a PRECISE radiological progression score for the likelihood of clinically significant change over time. We aimed to compare the PRECISE score with clinical progression in patients who are managed using an MRI-led AS protocol.

**Methods:**

A total of 553 patients on AS for low- and intermediate-risk PCa (up to Gleason score 3 + 4) who had two or more MRI scans performed between December 2005 and January 2020 were included. Overall, 2161 scans were retrospectively re-reported by a dedicated radiologist to give a PI-RADS v2 score for each scan and assess the PRECISE score for each follow-up scan. Clinical progression was defined by histological progression to ≥ Gleason score 4 + 3 (Gleason Grade Group 3) and/or initiation of active treatment. Progression-free survival was assessed using Kaplan-Meier curves and log-rank test was used to assess differences between curves.

**Results:**

Overall, 165/553 (30%) patients experienced the primary outcome of clinical progression (median follow-up, 74.5 months; interquartile ranges, 53–98). Of all patients, 313/553 (57%) did not show radiological progression on MRI (PRECISE 1–3), of which 296/313 (95%) had also no clinical progression. Of the remaining 240/553 patients (43%) with radiological progression on MRI (PRECISE 4–5), 146/240 (61%) experienced clinical progression (*p* < 0.0001). Patients with radiological progression on MRI (PRECISE 4-5) showed a trend to an increase in PSA density.

**Conclusions:**

Patients without radiological progression on MRI (PRECISE 1-3) during AS had a very low likelihood of clinical progression and many could avoid routine re-biopsy.

**Key Points:**

*• Patients without radiological progression on MRI (PRECISE 1–3) during AS had a very low likelihood of clinical progression and many could avoid routine re-biopsy.*

• *Clinical progression was almost always detectable in patients with radiological progression on MRI (PRECISE 4–5) during AS.*

*• Patients with radiological progression on MRI (PRECISE 4–5) during AS showed a trend to an increase in PSA density.*

**Electronic supplementary material:**

The online version of this article (10.1007/s00330-020-07256-z) contains supplementary material, which is available to authorized users.

## Introduction

Most policy groups around the world now recommend active surveillance (AS) as an appropriate option for patients with low-risk prostate cancer (PCa) [1, 2]. However, the current position is less than satisfactory as most of the evidence base to support its use is from single centres, no standard AS protocol exists and we lack a method of reliably ascertaining when true progression (by either grade or volume) occurs.

Multi-parametric MRI (mpMRI) has provided us with an opportunity to move away from random prostate biopsies and address the challenge of discriminating true progression from mere re-classification [3]. Recent evidence suggests that pre-biopsy MRI should be performed before confirmatory systematic transrectal ultrasound (TRUS)–guided biopsies during AS, together with MRI-targeted biopsies when indicated [4]. The ASIST trial initially showed no difference in the upgrade rate between patients having standard re-biopsy and those having MRI with 2 cores targeted to a lesion (upgrade to Gleason Grade Group 2 (GGG 2) was 21% vs 23% respectively, *p* = 0.9), although there were marked differences between the study centres [5]. However, after 2 years of follow-up, the authors found that baseline mpMRI before confirmatory biopsy resulted in 50% fewer failures of surveillance and less progression to higher-grade cancer, confirming the value of mpMRI during AS [6].

Addressing the serial use of MRI in AS, there are a small number of studies at present [7–11] and there is a lack of standardised reporting in serial MRI data across cohorts [12].

Following the standardisation work initiated with the Prostate Imaging Reporting and Data System (PI-RADS) guidelines, the Prostate Cancer Radiological Estimation of Change in Sequential Evaluation (PRECISE) panel was convened in 2016 [13]. The panel included ten experts in urology, eight in radiology and one in radiation oncology from the UK, Europe and North America and their objective was to define the conduct and reporting standards for patients on AS having serial MRI scans. A set of 394 statements relevant to prostate MRI reporting during AS was scored for agreement on a 9-point scale by each panelist individually prior to the meeting. All scores were collated and consensus (or lack of consensus) for each statement was assessed using the RAND/UCLA criteria. Each statement was then discussed and rescored anonymously by each panelist during the face-to-face meeting, and the PRECISE checklist and case report template for reporting prostate MRI during AS were developed according to the consensus reached. The key features of the PRECISE recommendations are the measurement of each lesion at every time point, and a determination of the likelihood of radiological progression using a 1-to-5 Likert scale (PRECISE score) as shown in Table 1.

**Table 1:** Assessment of likelihood of radiological progression on MRI in patients on active surveillance (PRECISE score)

PRECISE score	Assessment of likelihood of radiological progression	Example
1	Resolution of previous features suspicious on MRI	Previously enhancing area no longer enhances
2	Reduction in volume and/or conspicuity of previous features suspicious on MRI	Reduction in size of previously seen lesion that remains suspicious for clinically significant disease
3	Stable MRI appearance: no new focal/diffuse lesions	Either no suspicious features or all lesions stable in size and appearance
4	Significant increase in size and/or conspicuity of features suspicious for prostate cancer	Lesion becomes visible on diffusion-weighted imaging; significant increase in size of previously seen lesion
5	Definitive radiologic stage progression	Appearance of extracapsular extension, seminal vesicle involvement, lymph node involvement, or bone metastasis

Legend - PRECISE: Prostate Cancer Radiological Estimation of Change in Sequential Evaluation; MRI: Magnetic Resonance Imaging. Reprinted from Moore CM, Giganti F, Albertsen P, et al (2017) Reporting magnetic resonance imaging in men on active surveillance for prostate cancer: the PRECISE Recommendations - a report of a European School of Oncology Task Force. European Urology, 71(4) 648–655. Copyright (2017), with permission from Elsevier (https://www.europeanurology.com)

At present, there is limited literature on the application of the PRECISE recommendations in a clinical setting [14–17].

We report the application of the PRECISE recommendations in a large AS cohort of patients with serial MRI**.**

## Materials and methods

**Fig. 1:**
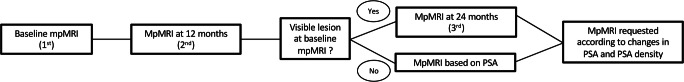
Overview of the active surveillance MR protocol at University College London Hospital. Legend - MpMRI: multiparametric magnetic resonance imaging; PSA: prostate specific antigen

At University College London Hospital, we have a cohort of > 620 patients who have undertaken MRI-guided AS. The cohort was established in 2005 in a prospective manner and is defined by patients who have had a prostate MRI and a biopsy-confirmed low- to intermediate-risk PCa (i.e. Gleason ≤ 3 + 4 and prostate-specific antigen (PSA) ≤ 20 ng/ml) as per UK National Institute for Health and Care Excellence (NICE) guidelines [18], and who have chosen AS as their initial management option. No maximum cancer core length or number of positive cores was stipulated for eligibility, due in part to the extensive use of targeted biopsies that can result in ‘risk inflation’ and exclude patients from AS unnecessarily. We also note that the number of involved cores is not part of disqualification criteria from AS according to recent European and UK NICE guidelines [19, 20]. At our institution, all clinical records and MR images are routinely reviewed as part of an audit performed for the internal evaluation of the AS service (Fig. 1) and no institutional review board approval was required [21].

In detail, the timing of MRI on AS at our centre is based on both baseline risk (i.e. presence of a visible lesion at mpMRI) and PSA/PSA density changes during follow-up. Biopsy recommendations are based either on the suspicion of progression on MRI, or on adverse PSA kinetics without MRI changes. All biopsies are performed using a transperineal approach.

Data are updated to February 15, 2020.

### Primary outcome

The primary outcome was to assess the relationship of the PRECISE score with clinical progression (defined by histological progression to ≥ GGG3 and/or initiation of active treatment).

### Secondary outcome

The secondary outcome was to evaluate the relationship between PSA density and PRECISE score.

### MRI protocol

All scans were performed according to international guidelines [22, 23]. Three different scanners were used: two 1.5-T (Symphony or Avanto, Siemens) and one 3-T system (Achieva, Philips), with a pelvic phased-array coil. The protocol comprised T2-weighted, diffusion-weighted (including long *b* sequences: 1400 s/mm^2^ for 1.5-T or 2000 s/mm^2^ for 3-T scanners) and dynamically contrast-enhanced imaging, as shown in Supplementary Table 1.

### MRI analysis and PRECISE score assessment

For each patient, all scans were retrospectively re-reported by a dedicated expert radiologist (F.G.) with 7 years of experience in prostate cancer imaging (reporting > 1800 prostate MRI scans/year) who had been actively involved in the writing of the PRECISE recommendations [13]. As per PRECISE recommendations, the radiologist was privy to PSA and initial biopsy results but blinded to the original MRI reports. The radiologist used a dedicated reporting tool (MIM® Symphony Dx v. 6.8.3) that provides a customised workflow that leads the user to report according to the PRECISE recommendations using a step-by-step procedure, allowing the comparison of data from serial scans [24].

Each visible lesion was scored according to PI-RADS v.2 guidelines at each time point [23]. At the second and subsequent scans, the radiologist assessed the PRECISE score for the likelihood of radiological progression from the last scan, using different MR features (conspicuity, increase in volume, signs of extracapsular extension or seminal vesicle invasion, etc.). A PRECISE score of 1 or 2 denotes radiological regression, a PRECISE 3 score indicates stability and a PRECISE 4 or 5 implies radiological progression (Table 1) [13].

As per PRECISE recommendations, the highest likelihood of clinically significant cancer of all separate lesions in a single scan provides the likelihood of clinically significant cancer for the whole prostate. Therefore, in the case of multifocal disease, the index lesion included in the analysis was the lesion with the highest PI-RADS score, and where there was more than one lesion at that score, the lesion with the highest volume was deemed the index lesion.

As there are still no explicit recommendations on which PRECISE score should be considered the most representative when multiple scans are acquired, we used the highest PRECISE score for each patient during the study period in this analysis. [14, 15]

We applied the following interpretation to the PRECISE recommendations:

i) PRECISE 3: for those scans with a visible lesion showing stable MR features over time, or a persistent negative scan.
ii) PRECISE 4: for a new lesion in a previous negative scan or if a lesion was not visible at baseline MRI but appeared on a subsequent scan and then had stable MR features over time (i.e. in this case the highest PRECISE score was the most representative, as a new lesion developed).

### Statistical analysis

Continuous variables were summarised by medians and interquartile ranges and the statistical significance of their differences was assessed using Mann-Whitney test. Categorical variables were summarised by frequencies and percentages. Wilcoxon test was applied to determine the statistical significance of differences.

The study asks whether there is an association between the PRECISE score and clinical progression. Because the PRECISE score could change over time with each successive scan at different time points, we included a time-dependent covariate for the PRECISE score using a Cox model predicting disease progression. Time zero was the date of the first biopsy showing PCa.

Progression-free survival was assessed using Kaplan-Meier curves and log-rank test was used to assess differences between curves.

The interaction term between each follow-up MRI and the corresponding PRECISE score, both considered time-dependent covariates, was tested to explore the variation of PSA density over time according to PRECISE score using linear regression and locally weighted scatterplot smoothing.

Statistical analyses were performed using R software (Version 3.4.2; Foundation for Statistical Computing).

All tests were two-sided, and *p* < 0.05 was considered statistically significant.

## Results

**Fig. 2:**
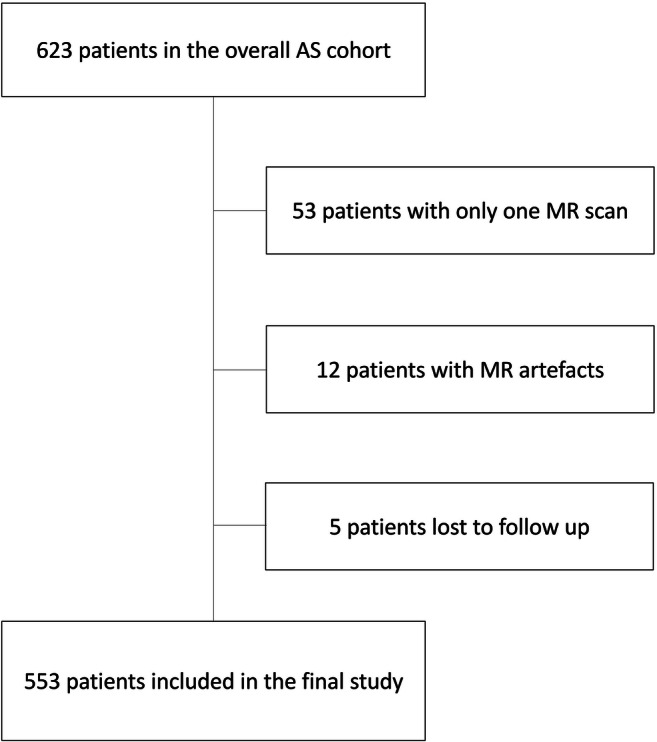
Flowchart shows study enrolment. Legend - AS: active surveillance; MR: magnetic resonance

Our final cohort comprised a total of 553 patients on AS with two or more prostate MR scans, all of diagnostic quality (Fig. 2).

All scans (*n* = 2161) had been performed between December 2005 and January 2020.

Overall, 232/553 (42%) patients were exclusively scanned on a 1.5-T and 8/553 (1.4%) exclusively on a 3-T scanner. Supplementary Table 2 shows the number of patients and MR scans included in the study.

Table 2 shows baseline and follow-up characteristics of our population. Overall, 306/553 (55%) patients had at least an additional biopsy, 178 (58%) of which were targeted by visual registration. All follow-up biopsies were performed using a transperineal approach.

**Table 2:** Descriptive statistics of all patients included in the study and stratified by biopsy progression, which is defined as one step in Gleason score upgrade (including those men with Gleason 3 + 3 at entry and subsequent Gleason 3 + 4)

	Overall (*n* = 553)	No biopsy progression (*n* = 177)	Biopsy progression (*n* = 129)	*p*
Age at diagnosis (years)	62 (56–67)	62 (57–66)	62 (56–67)	0.60
PSA at baseline MR (ng/ml)	6.3 (4.7–8.4)	6.51 (4.88–8.30)	6 (4.7–8.6)	0.47
Prostate volume at baseline MR (cc)	45.8 (32.7–63.4)	47.93 (36.15–64.38)	38.32 (29.3–56.7)	< 0.01
PSA density at baseline MR (ng/ml/ml)	0.12 (0.09–0.2)	0.12 (0.09–0.17)	0.15 (0.1–0.21)	0.01
Gleason score at entry				0.02
3 + 3	445 [80]	136 [77]	119 [92]	
3 + 4	108 [20]	41 [23]*	10 [8]	
Biopsy type at entry				-
Transperineal template	89 [16]	18 [10]	9 [7]	
Transperineal + targeted	76 [14]	11[6]	13 [10]	
Systematic	330 [60]	128 [72]	93 [72]	
Systematic + targeted	35 [6]	12 [7]	8 [6]	
TURP	23 [4]	8 [5]	6 [5]	
Baseline PI-RADS score				< 0.01
1–2	266 [48]	98 [56]	45 [35]	
3	104 [19]	36 [20]	23 [18]	
4	164 [30]	36 [20]	54 [42]	
5**	19 [3]	7 [4]	7 [5]	
Overall PRECISE score				< 0.01
1	100 [18]	43 [24]	4 [3]	
2	23 [4]	9 [5]	1 [1]	
3 (non-visible lesion)	152 [28]	50 [28]	5 [4]	
3 (visible lesion)	38 [7]	13 [7]	10 [8]	
4	211 [38]	56 [33]	94 [73]	
5	29 [5]	6 [3]	15 [11]	

Data are medians and interquartile range (parentheses); percentages in brackets [%]. Legend - PSA: prostate-specific antigen; MR: magnetic resonance; TURP: transurethral ultrasound resection of the prostate; PI-RADS: Prostate Imaging Reporting and Data System; PRECISE: Prostate Cancer Radiological Estimation of Change in Sequential Evaluation

*18/41 [44%] patients discontinued AS: 16/18 [89%] patients showed radiological progression (fourteen PRECISE 4 and two PRECISE 5), fourteen of which showed also PSA progression. The remaining 2/18 [11%] patients showed PSA progression but no radiological progression

**In the overall population, 13/19 [68%] patients had Gleason 3 + 3 and 6/19 [32%] patients had Gleason 3 + 4 at entry biopsy. In the ‘no biopsy progression’ group, 4/7 [57%] patients had Gleason 3 + 3 and 3/7 [43%] patients had Gleason 3 + 4 at entry biopsy. In the ‘biopsy progression’ group, all patients (7/7; 100%) had Gleason 3 + 3 at entry biopsy. In terms of lesion location, 4/19 [21%] were anterior lesions (two left anterior, one right anterior, one midline anterior) and the other 15/19 [79%] lesions were in the peripheral zone (8 on the left and 7 on the right)

For patients with baseline PI-RADS 4 and 5 lesions (*n* = 183), 133/183 (73%) had Gleason 3 + 3 and 50/183 (27%) had Gleason 3 + 4 at entry biopsy. Of them, 49/183 (27%) had a targeted biopsy at entry.

Table 3 lists the histopathological data of the whole population stratified by PRECISE score. If we have a closer look at those patients classified as PRECISE 4 (*n* = 211), 141/211 (67%) developed a new lesion, 36 of which (26%) were upgraded on histology, whilst 70/211 (33%) showed an increase in lesion size or conspicuity from baseline MRI, 58 of which (83%) were upgraded on histology. More in detail, if we focus on those patients who showed biopsy progression in the PRECISE 4 group (94/211; 45%), 36/94 (38%) developed a new lesion whilst 58/94 (62%) showed an increase in lesion size or conspicuity from baseline MRI. In this subcohort (*n* = 94), the median baseline PSA was 5.9 ng/ml (4.45–8.55) and the median baseline PSA density was 0.15 ng/ml/ml (0.11–0.21).

**Table 3:** Histopathological data of the whole population according to PRECISE score

		Number of biopsies			GS at entry		Upgrade	
		Only diagnostic biopsy	At least one follow-up biopsy		3 + 3	3 + 4	Upgrade to GS 3 + 4	Upgrade to GS ≥ 4 + 3
PRECISE 1–2 (*n* = 123)		66	57	Biopsy progression (*n* = 5)	5	0	4	1
				No biopsy progression (*n* = 52)	49	3	0	0
PRECISE 3 (*n* = 190)	Stable visible lesion (*n* = 38)	15	23	Biopsy progression (*n* = 10)	9	1	8	2
				No biopsy progression (*n* = 13)	9	4	0	0
	Non- visible lesion (*n* = 152)	97	55	Biopsy progression (*n* = 4 )	4		4	0
				No biopsy progression (*n* = 51 )	43	8	0	0
PRECISE 4 (*n* = 211)		61**	150	Biopsy progression (*n* = 94)	91*	3	80	14
				No biopsy progression (*n* = 56)	34*	22	0	0
PRECISE 5 (*n* = 29)		8***	21	Biopsy progression (*n* = 15)	10	5	7	8
				No biopsy progression (*n* = 6)	3	3	0	0

Legend - PRECISE: Prostate Cancer Radiological Estimation of Change in Sequential Evaluation; GS: Gleason score

*Of the 125 patients who showed baseline Gleason 3 + 3 prostate cancer, 63/125 (50%) had a PI-RADS 4 lesion and 10/125 (8%) a PI-RADS 5 lesion at baseline  magnetic resonance imaging **Sixty-one patients with a PRECISE score of 4 had no additional biopsy, and 26 progressed directly to active treatment. This was radical prostatectomy in 8, focal therapy in 8, radiotherapy in 5 and hormonal therapy in 3. The other two patients were put on watchful waiting

***Eight patients with a PRECISE score of 5 had no additional biopsy, and 5 progressed directly to active treatment. This was focal therapy in 2 and radiotherapy in 3. The other three patients had stage progression to T2a to radiological T3a and have chosen to continue with conservative management

### Primary outcome

The association between PI-RADS score at baseline and the PRECISE score at follow-up scans is summarised in Table 4. In summary, PI-RADS 1–2 tended to be associated with PRECISE scores ≤ 3 whilst patients with higher PI-RADS baseline scores (i.e. PI-RADS 4–5) had more than a two-thirds chance of being attributed a PRECISE score 4 or 5.

**Table 4:** Relationship between baseline PI-RADS score and the highest PRECISE score from scans for each patient in the overall population (*n* = 553)

	PI-RADS 1–2	PI-RADS 3	PI-RADS 4–5	Total
PRECISE 1–2	34	55	34	123
PRECISE 3	144	22	24	190
PRECISE 4–5	88	27	125	240
Total	266	104	183	553

Legend - PI-RADS: Prostate Imaging Reporting and Data System; PRECISE: Prostate Cancer Radiological Estimation of Change in Sequential Evaluation

**Fig. 3:**
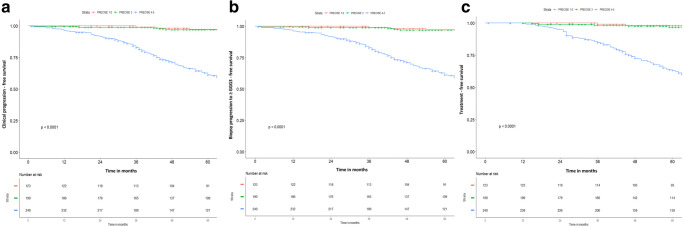
Kaplan-Meier curves showing the rate of clinical progression (≥ Gleason Grade Group 3 and initiation of active treatment) (**a**), only biopsy progression (≥ Gleason Grade Group 3) (**b**) and only initiation of active treatment (**c**) stratified by PRECISE score (1–2 vs 3 vs 4–5) in the overall population. Legend - GGG: Gleason Grade Group

Median follow-up of the overall population was 76 months (52–100.5). In Fig. 3 a, we illustrate the proportion of patients that were free of clinical progression at 12, 24 and 60 months. Overall, 165/553 (30%) patients experienced the primary outcome of clinical progression, with a median follow-up of those without clinical progression of 74.5 months (53–98), as listed in Supplementary Table 3 and Supplementary Table 4.

For PRECISE 1–2, freedom from clinical progression was 100% at 12 and 24 months and 97% at 60 months. For PRECISE 3, freedom from clinical progression was 99% at 12 and 24 months and 97% at 60 months and for PRECISE 4–5 it was 96%, 91% and 61%, respectively. There was a significant difference in clinical progression between PRECISE 1–2 vs 4–5 and PRECISE 3 vs 4–5 (*p* < 0.001).

The proportion of patients free from histological progression to ≥ GGG 3 or initiation of active treatment (analysed separately) is shown in Fig. 3 b and c, respectively.

The Cox model predicting disease progression with a time-dependent covariate for the PRECISE score is shown in Supplementary Table 5. Supplementary Table 6 shows the number of patients with biopsy progression stratified according to a negative MR scan or radiological progression before biopsy.

### Secondary outcome

**Fig. 4:**
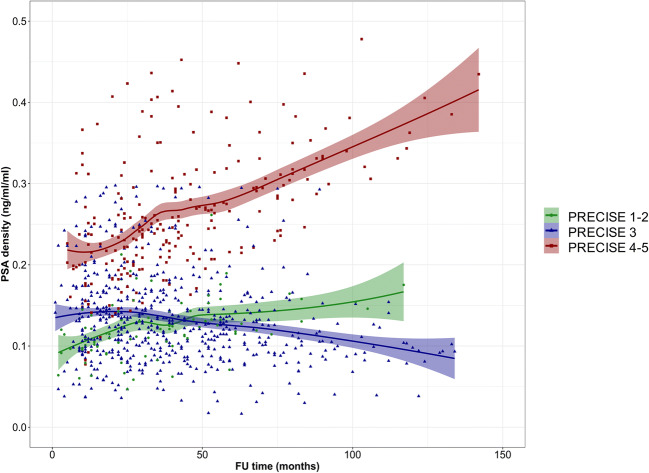
Multivariable relationship between each follow-up MRI and PSA density over time stratified by PRECISE score (1–2 vs 3 vs 4–5) in the overall population. Legend - PSA, prostate specific antigen; FU: follow-up

There was a relationship between change in PSA density and the PRECISE score. The interaction term between the PRECISE score and each follow-up MRI in predicting PSA density variation over time was significant for PRECISE 4–5 (hazard ratio 1.04; *p* = 0.01) (Fig. 4 and Supplementary Table 7).

Table 5 reports the quantitative and qualitative parameters used to assess the PRECISE score for each patient included in the study in accordance with the PRECISE case report form.

**Table 5:** Quantitative and qualitative parameters used to assess the PRECISE score for each patient included in the study in accordance with the PRECISE case report form

	PRECISE 1 (*n* = 100)	PRECISE 2 (*n* = 23)	PRECISE 3 without lesion (*n* = 152)	PRECISE 3 with visible lesion (*n* = 38)	PRECISE 4 (*n* = 211)	PRECISE 5 (*n* = 29)
PSA (ng/ml)	6.60 [4.68–9]	7 [3.6–11]	6.4 [3.82–9.61]	6.53 [4.05–10]	7.40 [5.17–10]	9 [7.1–13.36]
PSA density (ng/ml/ml)	0.11 [0.07–0.16]	0.11 [0.07–0.19]	0.11 [0.07–0.15]	0.09 [0.07–0.14]	0.15 [0.1–0.23]	0.22 [0.10–0.40]
Prostate volume on T2-WI (cc)	54.45 [42.60–74]	53.63 [39.96–67.14]	52.66 [36.59–73.26]	68.91 [32.86–82.93]	43.84 [33.19–63.75]	40.23 [29.79–69.95]
Magnet strength						
1.5 T	76	16	129	32	164	22
3 T	24	7	23	6	47	7
Likert score						
1–2	100	–	132	–	–	–
3	–	21	20	13	43	2
4	–	2	–	23	114	9
5	–	–	–	2	54	18
PI-RADS score						
1–2	100	2	146	–	–	–
3	–	20	6	14	40	2
4	–	1	–	22	137	16
5	–	–	–	2	34	11
T3a–T3b	–	–	–	–	–	29
Single max diameter (cm)	–	0.77 [0.60–1.23]	–	0.82 [0.66–1.23]	0.92 [0.67–1.38]	1.37 [1.01–2.28]
Biaxial measurement (cm^2^)	–	0.73 [0.38–1.05]	–	0.75 [0.5–1.12]	0.77 [0.44–1.27]	1.22 [0.84–2.49]
Lesion volume (ellipsoid formula) on T2-WI (cc)	–	0.12 [0.10–0.19]	–	0.23 [0.14–0.33]	0.22 [0.11–0.54]	0.45 [0.24–1.57]
Lesion volume (planimetry) on T2-WI (cc)	–	0.14 [0.09–0.21]	–	0.21 [0.13–0.29]	0.23 [0.12–0.49]	0.45 [0.23–1.1]
Sequence where lesion best seen:						
T2-WI	–	11	–	10	63	12
DWI	–	5	–	11	70	6
DCE	–	7	–	17	78	11
Lesion volume (where lesion best seen)	–	0.15 [0.12–0.27]	–	0.21 [0.16–0.37]	0.30 [0.15–0.56]	0.68 [0.26–1.59]
ADC min (× 10^−3^ mm^2^/s)	–	0.42 [0.22–0.74]	–	0.48 [0.32–0.69]	0.47 [0.26–0.64]	0.40 [0.27–0.53]
ADC mean (× 10^−3^ mm^2^/s)	–	0.89 [0.81–1.10]	–	0.84 [0.70–0.97]	0.84 [0.69–0.99]	0.80 [0.71–0.98]
ADC median (× 10^−3^ mm^2^/s)	–	0.91 [0.80–1.12]	–	0.86 [0.70–1.01]	0.83 [0.67–1]	0.79 [0.71–0.95]
Lesion location						
Right peripheral zone	–	9	–	16	100	10
Left peripheral zone	–	13	–	14	82	14
Right transitional zone	–	1	–	4	15	3
Left transitional zone	–	–	–	4	14	2
Parameters changed from previous scan:						
Dimensional criteria	–	12	–	–	62	–
Conspicuity:						
T2-WI	–	4	–	–	5	–
DWI	–	1	–	–	2	–
DCE	–	2	–	–	1	–
New lesion	–	–	–	–	141	–
PI-RADS score	–	4	–	–	–	–
Stage progression	–	–	–	–	–	29

Data are medians with interquartile ranges in brackets Legend - PSA: prostate specific antigen; T2-WI: T2-weighted imaging; PI-RADS: Prostate Imaging Reporting and Data System; DWI: diffusion-weighted imaging; DCE: dynamic contrast-enhanced; ADC: apparent diffusion coefficient

**Fig. 5:**
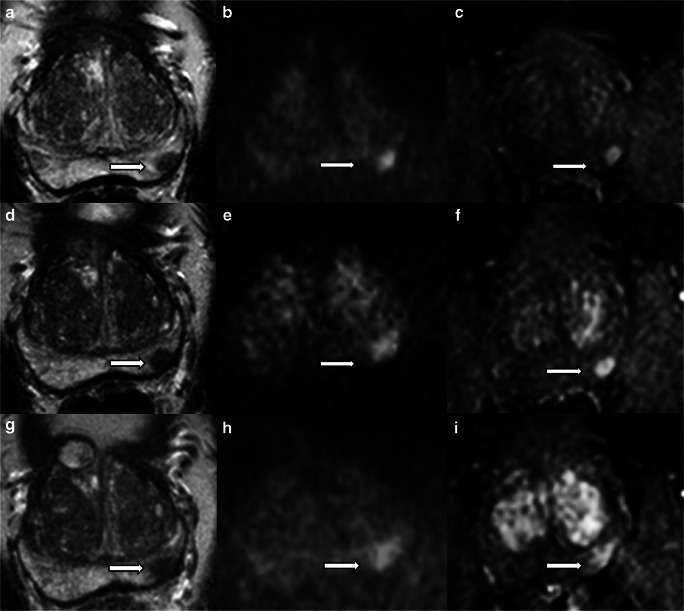
Fifty-year-old man on active surveillance for Gleason 3 + 3 (4 mm) prostate cancer in the left peripheral zone on standard transrectal ultrasound biopsy. The mpMRI scan at baseline (**a**, **b**, **c**) shows a left-sided peripheral zone lesion (arrows) between 4 and 5 o'clock characterised by low signal intensity on T2-weighted imaging (**a**), restricted diffusion on diffusion-weighted imaging (**b**) and early enhancement on dynamic contrast-enhanced imaging (**c**). The lesion showed progressive radiological change in volume and conspicuity after one (**d**, **e**, **f**) and two (**g**, **h**, **i**) years (PRECISE score: 4). The patient was treated with high-intensity focused ultrasound

Figure 5 shows a case classified as PRECISE 4.

## Discussion

This is the first report that applies the PRECISE criteria for radiological progression in a UK cohort of patients with PCa on an MRI-led AS programme.

We have shown that radiological stability is associated with 97% freedom from clinical progression at 5 years. We have also demonstrated that a PI-RADS score of 4 or 5 at entry to surveillance is associated with PRECISE 4 or 5 in up to 68% at a median of 5 years—that is, more than two-thirds of visible lesions on MRI will increase in size, conspicuity or stage over a 5-year period.

Additionally, an increase in PSA density over time is significantly associated with radiological progression (PRECISE 4–5).

The most appropriate definition of progression in patients on AS is a challenging one. Whilst inclusion of patients with low-volume GGG 2 is increasing, it is also sometimes stated as a threshold for initiation of active treatment. We chose GGG 3 as the histological threshold for clinical progression, as this fits with our clinical practice of recommending treatment in all of these patients. Some patients, particularly those who have GGG 2 at enrolment on AS, and who show radiological progression, wish to avoid a biopsy and proceed with active treatment. As they had already met the criteria for choosing active treatment at baseline, this approach was permitted.

We therefore chose a combined endpoint of either histological progression to GGG 3, or initiation of active treatment as the definition of clinical progression.

In our MRI-led AS programme, biopsies are not offered on a protocol basis. Instead they are offered to patients ‘for cause’. The triggers for the recommendation comprise adverse changes in MRI or PSA kinetics. This approach was developed in response to our work in this field, where we demonstrated a high negative predictive value of MRI for GGG 3 disease. Whilst this protocol differs from all other published protocols, we know that in practice, compliance rates with protocol biopsy can be as low as 20 to 30%, in both routine practice and formal studies such as PRIAS [25], and actual biopsy rates may be more similar in the two approaches than the protocols would suggest [26, 27].

Whilst some would see the lack of additional biopsy as a drawback, we believe that it has significant advantages in terms of patient acceptability and compliance, and that the risk of ‘loss of opportunity for cure’ is no higher using this approach than standard biopsy alone. For example, in our cohort, 1/553 patients (< 1%) developed nodal disease and 2/553 (< 1%) patients had bone metastases whilst on surveillance, with median follow-up of 6.3 years. This compares well to the Sunnybrook cohort [28], where 13/980 (1.3%) patients developed lymph node disease, and 18/980 (1.8%) patients developed bone metastases, with a median follow-up of 6.3 years.

### Limitations

The first, and possibly the most relevant, limitation is that the entry biopsy was often TRUS-guided and without a clear definition of the lesion location, whilst subsequent biopsies were done using a transperineal approach and this could represent a possible bias. Since this is an image-guided AS cohort, not all patients underwent re-biopsy during follow-up and resampling was often triggered by apparent tumour growth on mpMRI. As this is a retrospective study, this means that patients with negative mpMRI results did not routinely undergo biopsies and we acknowledge that this factor could contribute to verification bias [29].

However, we know that the likelihood of clinically significant PCa with negative MRI is low and such patients are unlikely to benefit from a biopsy [30].

We should also mention that patients entering AS at our centre with a diagnostic biopsy at another centre are required to have a concordant mpMRI and biopsy and undergo repeat biopsy to assess any discordance if required.

We acknowledge that this is a single-centre study in a centre with significant prostate mpMRI experience, and the results may not be generalisable to all centres.

Only a single radiologist, although highly experienced in prostate MR reporting, applied the PRECISE score in the whole cohort. However, we have recently demonstrated the high interobserver agreement of the PRECISE score between two expert radiologists in a multicentre study [14].

The retrospective assessment of the PRECISE score could be seen as another limitation, as we have been using the PRECISE score only from 2016 onwards but the assessment of radiological progression before that date was subjective and not well-standardised. However, after completion of the study, we observed that > 95% of the original reports were in line with the PRECISE scores that were retrospectively assessed, corroborating our current results.

Lastly, MRI quality has improved over time, and the earliest scans might be less informative than the most recent ones. Some patients received scans on both 1.5-T and 3-T scanners, and this may have limited the ability to accurately compare measurements of lesions between scans.

### Implications for AS programmes

AS programmes typically have more intense follow-up in the early period, to identify patients who were misclassified. There has long been controversy over the use of PSA kinetics in patients on AS, with some reports suggesting that PSA kinetics are not predictive for biopsy upgrade [31].

In this cohort, we demonstrate an association of change in PSA density with radiological progression. If these findings are confirmed in larger cohorts, we may be able to use PSA density as a trigger for further investigation rather than a protocol-based approach using time from diagnosis.

## Conclusions

Our study suggests that the widespread use of the PRECISE recommendations might have two main clinical consequences: (i) identification of patients on AS who progress (PRECISE 4–5) in a timely manner, promoting re-biopsy/treatment; (ii) avoidance of repeat biopsy (and a lower intensity surveillance schedule) for patients with PRECISE 1–3, reducing the burden of surveillance for the individual and the healthcare system.

We do not promote the adoption of our AS protocol in every clinical setting, but we provide here the first application of the PRECISE recommendations in a cohort of patients monitored according to the UK NICE guidelines.

Future randomised studies (e.g. a study design of ‘biopsy’ versus ‘no biopsy’ according to PRECISE, with presence or absence of clinical progression as the ultimate reference) will be more informative.

We acknowledge that the PRECISE score will require further validation in prospective multicentre cohorts, using a range of reporters. Further analysis in larger cohorts, including the Movember GAP3 cohort of over 15,000 patients on AS across 25 countries, is planned [2].

## Electronic supplementary material

ESM 1 (DOCX 27 kb)

## Abbreviations

AS: Active surveillance
DCE: Dynamic contrast enhanced
DWI: Diffusion-weighted imaging
GGG: Gleason Grade Group
MRI: Magnetic resonance imaging
NICE: National Institute for Health and Care Excellence
PCa: Prostate cancer
PI-RADS: Prostate Imaging Reporting and Data System
PRECISE: Prostate Cancer Radiological Estimation of Change in Sequential Evaluation
PSA: Prostate-specific antigen
T2-WI: T2-weighted imaging
TRUS: Transrectal ultrasound
