# Supplementary material for: Natural history of prostate cancer on active surveillance: stratification by MRI using the PRECISE recommendations in a UK cohort
Source: Eur Radiol. 2020 Sep 30;31(3):1644–55. doi: 10.1007/s00330-020-07256-z (PMC7880925; doi:10.1007/s00330-020-07256-z)
Supplement: Supplementary file 1 — (DOCX 27 kb) [file 330_2020_7256_MOESM1_ESM.docx]

**Supplementary Table 1:** MR imaging parameters at our Institution

|  | 1.5T | 3T |
| --- | --- | --- |
| T2-weighted imaging (axial) | | |
| Sequence | TSE | TSE |
| Field of view (mm) | 200 | 180 |
| Slice thickness (mm) | 3 | 3 |
| Matrix size | 256 x 256 | 300 x 300 |
| Pixel size (mm x mm) | 0.8 x 0.8 | 0.6 x 0.6 |
| TE (ms) | 95 | 100 |
| TR (ms) | 5340 | 5407 |
| Acquisition time | 4min 2sec | 5min 13sec |
| Diffusion-weighted imaging | | |
| Sequence | SSEPI | SSEPI |
| Field of view (mm) | 320 | 220 |
| Slice thickness (mm) | 5 | 5 |
| Matrix (read) | 172 x 172 | 168 x 168 |
| Pixel size (mm x mm) | 1.5 x 1.5 | 1.29 x 1.29 |
| TE (ms) | 101 | 80 |
| TR (ms) | 2200 | 2304 |
| Multiple *b*-values (s/mm^2^) | 0, 150, 500, 1000 | 0, 150, 500, 1000 |
| Acquisition time | 5min 44sec | 6min 15sec |
| High *b*-values (s/mm^2^) | 1400 | 2000 |
| Acquisition time | 3min 39sec | 2min6sec |
| Dynamic contrast enhanced | | |
| Sequence | 3D FLASH | T1-FFE |
| Field of view (mm) | 260 | 180 |
| Matrix (read) | 192 x 192 | 140 x 162 |
| Pixel size (mm x mm) | 1.4 x 1.4 | 1.29 x 1.30 |
| TE (ms) | 2.50 | 2.8 |
| TR (ms) | 5.61 | 5.8 |
| Fat suppression | Fat sat | SPAIR |
| Acquisition time | 5min 43sec | 3min11sec |
| Temporal resolution (sec) | 13 | 13 |

Legend: TSE: Turbo Spin Echo; TE: echo time; TR: repetition time; SSEPI: single shot echo planar imaging; FLASH: Fast low angle shot; FFE: fast field echo; SPAIR: Spectral attenuated inversion recovery

**Supplementary Table 2:** Number of patients and MR scans included in the study

| **Number of MR scans** | **Number of patients (n = 553)** |
| --- | --- |
| **2** | **112 [20%]** |
| **3** | **141 [25%]** |
| **4** | **121 [22%]** |
| **5** | **104 [19%]** |
| **6** | **42 [8%]** |
| **7** | **18 [3%]** |
| **8** | **7 [1.4%]** |
| **9** | **5 [1%]** |
| **10** | **1 [0.2%]** |
| **11** | **1 [0.2%]** |
| **12** | **1 [0.2%]** |

Legend: Percentages in brackets [%].

The median number of scans per patient was 4 [IQR: 3-5].

The median interval time between baseline and the second scan was 14 months [IQR: 11-22].

**Supplementary Table 3:** List of the outcome and different treatments in the whole cohort.

|  | **Overall**  **(n=553)** |
| --- | --- |
| Outcome  No treatment  Active treatment  Watchful waiting | 374 [78]  165 [29]  14 [3] |
| Treatment *  Radical prostatectomy  Focal therapy  EBRT  Brachytherapy  ADT | 51/165 [31]  75/165 [45]  24/165 [15]  4/165 [2]  11/165 [7] |

Legend: Percentages in brackets [%]. EBRT = external beam radiotherapy; ADT = androgen deprivation therapy.

* Of the 165 patients who experienced clinical progression, 23 [14%] had biopsy upgrading (GGGS ≥ 3) and 142 [86%] were treated according to clinical decision making.

**Supplementary Table 4**: Indications for treatment in the 165 patients who experienced clinical progression.

|  | **Number of patients** |
| --- | --- |
| **Radiological progression** | **146 *** |
| **Only biopsy progression (GGG2)** | **7** |
| **Only biopsy progression (GGG3)** | **2** |
| **Only PSA/PSA density progression** | **10** |

* 94 and 21 of which showed also GGG2 and GG3 biopsy upgrade, respectively.

Legend – GGG: Gleason Grade Group; PSA: prostate specific antige.

**Supplementary Table 5**: Multivariable Cox regression model with PRECISE 4-5 as a time dependent covariate predicting ≥ Gleason Grade Group 3 and/or initiation of active treatment.

| Predictors | Multivariable analysis | |
| --- | --- | --- |
|  | HR (95% CI) | *p* value |
| Age | 0.99 (0.96-1.02) | 0.48 |
| Baseline PSA density (ng/ml/ml) | 13.12 (1.62-106.01) | 0.016 |
| PRECISE score |  |  |
| 1-3 | Ref. | - |
| 4-5 | 28.38 (8.06-99.95) | < 0.001 |
| Baseline Gleason score |  |  |
| 3+3 | Ref. | - |
| 3+4 | 0.99 (0.61 - 1.60) | 0.98 |
| Baseline PI-RADS  PI-RADS 2  PI-RADS 3  PI-RADS 4  PI-RADS 5 | Ref.  2.62 (1.44 - 4.74)  2.56 (1.51 - 4.35)  1.97 (0.79 - 4.86) | -  0.001  < 0.001  0.14 |

**Supplementary Table 6**: Number of patients with biopsy progression (n=129) who had a negative MR or showed MR progression before biopsy.

|  | Number of patients | Number of patients with ≥ Gleason 4+3 upgrade |
| --- | --- | --- |
| Negative MR before biopsy | 44 | 7 |
| MR progression before biopsy | 85 | 18 |
| Total | 129 | 25 |

**Supplementary Table 7**: Multivariable interaction analysis predicting PSA density variation over time between follow up-MRI and the corresponding PRECISE score. Covariates: age, baseline PSA density, PRECISE score, baseline Gleason score, baseline maximum cancer core length, baseline overall number of positive cores, baseline PI-RADS.

| Interaction predictor | Multivariable analysis | |
| --- | --- | --- |
|  | Interaction HR (95% CI) | *p* value |
| PRECISE score  PRECISE 1-2 * follow up MRI  PRECISE 3 * follow up MRI  PRECISE 4-5 * follow up MRI | Ref.  0.98 (0.97 – 0.99)  1.04 (1.03 – 1.06) | -  0.4  0.01 |
